# Choroidal Thickness in 3001 Chinese Children Aged 6 to 19 Years Using Swept-Source OCT

**DOI:** 10.1038/srep45059

**Published:** 2017-03-22

**Authors:** Shuyu Xiong, Xiangui He, Junjie Deng, Minzhi Lv, Jiali Jin, Sifei Sun, Chunxia Yao, Jianfeng Zhu, Haidong Zou, Xun Xu

**Affiliations:** 1 Department of Preventative Ophthalmology, Shanghai Eye Disease Prevention and Treatment Center, Shanghai Eye Hospital, Shanghai 200040, China; 2 Department of Ophthalmology, Shanghai General Hospital, Shanghai Jiao Tong University, Shanghai 200080, China; 3 School of Public Health, Fudan University, Shanghai 200032, China; 4 Jiading Center for Disease Prevention and Control, Shanghai 201901, China; 5 Songjiang Center for Disease Prevention and Control, Shanghai 201620, China

## Abstract

The purpose of the cross-sectional study is to describe the values and distribution of choroidal thickness and to explore its related factors, especially age, in Chinese children. A total of 3001 Chinese school children aged 6 to 19 years underwent comprehensive ophthalmic examinations, including axial length and cycloplegic refraction. Choroidal thickness was measured by swept-source optical coherence tomography (SS-OCT). There was a greater difference in the more central regions between the myopes and emmetropes. Multiple regression analysis was performed to determine the associated factors of choroidal thickness. The results demonstrated that age was independently positively related to choroidal thickness for emmetropes (β = 3.859, p < 0.001), and mild myopes with spherical equivalent greater than −2.00 D (−1.25 D < spherical equivalent ≤ −0.50 D: β = 3.476, p = 0.006; −2.00 D < spherical equivalent ≤ −1.25 D: β = 3.232, p = 0.020). However, no significant relationship between age and choroidal thickness was found in children with spherical equivalent ≤ −2.00 D, suggesting that the protective effect of physiologic choroidal growth with age against rapid axial elongation disappeared while axial elongation becomes the dominant determinant of choroidal thickness among children with myopia worse than −2.00 D.

The choroid is a highly vascularized structure between the retina and sclera with numerous functions in the eye, including the metabolic support of the retina, modulation of ocular temperature and intraocular pressure (IOP) and others^1^,^2^. Therefore, it could have a clinically important role in the pathophysiology of several choroidal vascular diseases such as age-related macular degeneration (AMD)^3^, polypoidal choroidal vasculopathy (PCV)^4^ and central serous chorioretinopathy^5^. In addition, the choroid regulates scleral extracellular matrix (ECM) remodeling, which can lead to changes in eye size and refraction, by relaying retina-derived signals to the sclera^1^,^6^. Thus, it may play a vital role in the homeostatic control of eye growth and consequently, in the etiology of myopia and hyperopia^1^.

Since the development of enhanced depth imaging optical coherence tomography (EDI-OCT)^7^ and more recently, swept-source optical coherence tomography (SS-OCT)^8^ which has enabled the *in vivo* imaging and measurement of the choroid, the characteristics of the choroid under normal^8^,^9^,^10^,^11^,^12^,^13^,^14^,^15^,^16^,^17^,^18^,^19^,^20^,^21^,^22^,^23^,^24^,^25^,^26^,^27^,^28^,^29^,^30^,^31^, as well as under certain pathologic conditions^32^,^33^,^34^,^35^,^36^,^37^, have been described. Age^8^,^9^,^10^,^11^,^17^, refractive error and axial length (AL)^8^,^11^,^13^,^14^,^17^,^38^,^39^,^40^ have been well established as major factors that can impact *in vivo* choroidal thickness in healthy adults, with increasing age, AL and decreasing refractive diopter being associated with a reduction of choroidal thickness. While the number of studies on choroidal thickness in healthy children has also increased in recent years, the factors that have been shown to affect choroidal thickness varied, especially with regards to age^19^,^20^,^21^,^22^,^23^,^24^,^25^,^26^,^27^,^28^,^29^,^30^,^31^. Age was found to be positively associated with subfoveal choroidal thickness in white children^20^,^24^,^25^,^26^,^27^, but negatively related to choroidal thickness in Asian children, where the prevalence of myopia was higher^21^,^22^,^23^,^29^,^30^. Some studies indicated that there was no relationship between age and choroidal thickness^19^,^31^. In addition, for the relationship between choroidal thickness and gender, it has been reported that girls have higher choroidal thickness or choroidal volume than boys in some studies^19^,^20^,^21^,^24^,^30^, although this did not reach statistical significance after adjusting for other related factors. However, one study found significantly thicker subfoveal choroidal thickness in boys than in girls^29^, while no relationship with gender was observed in other studies^26^,^27^,^31^.

The reasons for some of the contrary results obtained in previous studies may be due to the relatively small sample size^20^,^21^,^22^,^23^,^26^,^27^,^30^,^31^, limited range of ages^19^,^25^ or uneven proportions of refractive errors^24^. Thus, the purpose of this cross-sectional study is to investigate the characteristics of choroidal thickness and to explore the relationship between choroidal thickness and age in a large sample of normal Chinese school children aged 6 to 19 years with a wide range of refractive errors using SS-OCT.

## Results

### General Characteristics

Among the 3044 children enrolled in this study, 43 children were excluded, including 36 with amblyopia, 3 with strabismus and 4 with poor SS-OCT imaging quality. Consequently, a total of 3001 children aged 6 to 19 years with a mean age of 11.7 ± 3.4 years and 51.8% male were included in the final analysis. Among the 3001 children, the mean AL was 24.04 ± 1.31 mm (range from 20 to 29 mm) and the mean spherical equivalent was −1.20 ± 2.48 D (range from −11.38 D to +8.38 D). There were 1548 myopes, 441 emmetropes and 1012 hyperopes. The general characteristics of the enrolled participants are listed in Table 1.

### Topographical Variation of Choroid

**Figure 1:**
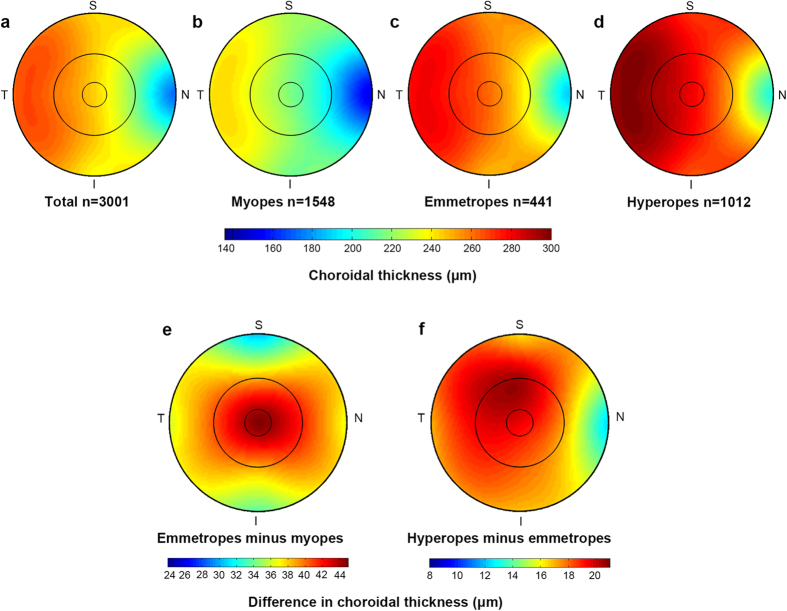
Topographical variation of choroid thickness in overall participants (**a**), myopes (**b**), emmetropes (**c**) and hyperopes (**d**). Average difference in choroidal thickness between emmetropes and myopes (**e**), as well as between hyperopes and emmetropes (**f**).

For all participants, the choroidal thickness differed significantly among the nine macular sectors of the ETDRS grid (p < 0.001). The mean central foveal choroidal thickness was 245 ± 66 μm and decreased from the temporal sector toward the nasal sector horizontally (all p < 0.001). It was thicker in the parafoveal sectors than that in the perifoveal sectors vertically (all p < 0.001, Fig. 1a). Similar topographical variations were observed in individuals with different refractive statuses (Fig. 1b,c and d).

In all of the nine sectors of the ETDRS grid, the choroid was thickest in hyperopes, followed by emmetropes, and was thinnest in myopes (all p < 0.001). As illustrated in Fig. 1e and f, the difference in choroidal thickness between emmetropes and myopes was greater in the more central regions compared to those in the more peripheral regions, while the difference between emmetropes and hyperopes was much smaller, with the smallest difference found in the perifoveal nasal sector.

### Association of choroidal thickness With Age and Gender

**Figure 2:**
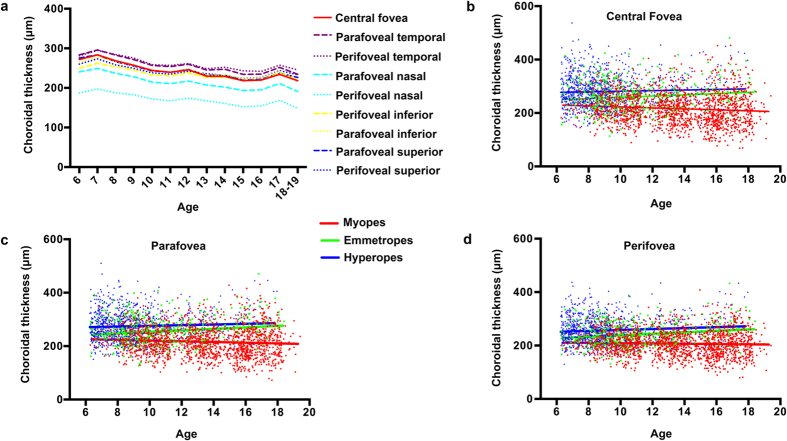
The association between average choroidal thickness and age for all participants in all of the nine sectors (**a**). Linear correlation between age and choroidal thickness under different refractive status in central fovea (**b**), parafovea (**c**), and perifovea (**d**).

The distributions of central foveal choroidal thickness in each age interval are displayed in Table 2. In general, the choroidal thickness was negatively associated with age in all of the nine sectors for the total population (Fig. 2a). However, for emmetropes, choroidal thickness was positively associated with age for the central fovea (r = 0.116, p = 0.015, Fig. 2b), parafovea (r = 0.157, p = 0.015, Fig. 2c), and perifovea (r = 0.181, p < 0.001, Fig. 2d). For hyperopes, there was a trend towards a positive relationship between age and choroidal thickness, but statistical significance was only observed for the perifovea (central fovea: r = 0.044, p = 0.158; parafovea: r = 0.055, p = 0.079; perifovea: r = 0.086, p = 0.006; Fig. 2b to d). For myopes, increasing age was significantly associated with a reduction of choroidal thickness in the central fovea (r = −0.093, p < 0.001, Fig. 2b) and parafovea (r = −0.076, p = 0.003, Fig. 2c), but not in the perifovea (r = −0.036, p = 0.162, Fig. 2d).

**Figure 3:**
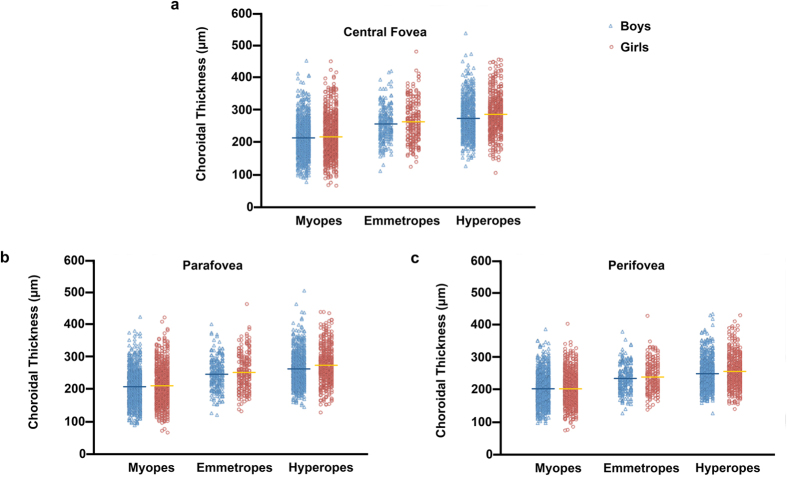
Choroidal thickness of girls and boys in central fovea (**a**), parafovea (**b**) and perifovea (**c**) for hyperopes, emmetropes and myopes.

There was no significant difference between genders for myopes and emmetropes (all p > 0.05 for central, parafovea and perifovea regions; Fig. 3). However, for hyperopes, girls had significantly thicker choroidal thickness than boys, with a greater mean difference in the more central regions (central fovea: mean difference = 13 μm, p = 0.001; parafovea: mean difference = 11 μm, p = 0.001; perifovea: mean difference = 7 μm, p = 0.022; Fig. 3).

### Multiple Regression Analysis

Among all the participants, AL, spherical equivalent, age, BMI and IOP were independently related to the central foveal choroidal thickness, with a determination coefficient (R^2^) of 0.269. According to the model, longer AL, more myopic diopters and younger age were significantly associated with thinner central foveal choroidal thickness (AL: β = −15.59; spherical equivalent: β = 7.645; age: β = 1.156; all p < 0.01).

In the multiple regression model, there was a strong positive relationship between age and central foveal choroidal thickness for emmetropes, but not for myopes and hyperopes (myopes: β = 1.091, p = 0.051;emmetropes: β = 3.859, p < 0.001; hyperopes: β = 1.569, p = 0.070, Table 3). However, after stratifying myopes, age was found to be positively associated with central foveal choroidal thickness in mild myopes, while no relationship was observed in high and moderate myopes (high myopes: β = −1.656, p = 0.243; moderate myopes: β = −0.265, p = 0.804; mild myopes: β = 2.416, p = 0.002, Table 4). Furthermore, after stratifying mild myopes, the groups −1.25 D < spherical equivalent ≤ −0.50 D and −2.00 D < spherical equivalent ≤ −1.25 D were found to have a positive relationship between age and central foveal choroidal thickness. However, in the group −3.00 D < spherical equivalent ≤ −2.00 D, the relationship between age and central foveal choroidal thickness was not found (−1.25 D < spherical equivalent ≤ −0.50 D: β = 3.476, p = 0.006; −2.00 D < spherical equivalent ≤ −1.25 D: β = 3.232, p = 0.020; −3.00 D < spherical equivalent ≤ −2.00 D: β = 0.799, p = 0.561, Table 4). These features were also observed for the parafoveal and perifoveal regions.

Although there was a tight relation between AL and spherical equivalent (r = −0.839, p < 0.001), no statistical significant collinearity (variance inflation factor ranged from 1.047 to 3.193) were found between AL and spherical equivalent on the variations in choroidal thickness in all the above regression models.

## Discussion

To our knowledge, to date, the present study represents the largest population of school-based children with the widest range of ages and refractive errors to assess the values, distribution pattern, and associated factors, especially age, for macular choroidal thickness. The results demonstrated that increasing age was associated with increased choroidal thickness for emmetropes and mild myopes with spherical equivalent greater than −2.00 D, while no association was observed for myopes equal to or less than −2.00 D. Among hyperopes, girls had a significantly thicker choroid than boys, but this significance no longer existed after adjusting for related factors.

Subfoveal choroidal thickness varies among individuals of different ethnicities. Studies on white populations reported choroidal thickness of 337 ± 82 μm in 104 Australian children aged 10 to 15 years and 341.96 ± 74.7 μm in 348 French children aged 3.5 to 14.9 years^25^,^26^, while those on Asian populations reported choroidal thickness of 260 ± 57.2 μm in 100 Japanese children aged 3 to 15 years and 251 ± 61 μm in 276 Chinese children aged 7 to 13 years^21^,^31^. In addition, choroidal thickness values could be partially affected by the instruments used and the measurement protocols. In the present study, SS-OCT and the ETDRS grid was adopted for the measurement of choroidal thickness, and the mean central foveal choroidal thickness of all participants was 245 ± 66 μm, which is in relatively close agreement with the values found in two previous studies on Asian children using the same method (260 ± 57.2 μm, aged 3 to 15 years; and 251 ± 61 μm, aged 7 to 13 years)^21^,^31^. The mean choroidal thickness measured in the present study was the thickest temporally and the thinnest nasally for all participants, as well as for those with different refractive status, which is consistent with previous pediatric studies^21^,^22^,^23^,^24^,^26^,^29^,^30^,^31^.

The choroid was thickest in hyperopes, followed by in emmetropes, and was thinnest in myopes, as previously demonstrated in studies on pediatric and adult populations^25^,^31^,^40^. Our study indicated a greater difference in the more central region between myopes and emmetropes and an evenly distributed difference across the macular region between hyperopes and emmetropes, except for the nasal sector, where the smallest difference was observed. It is speculated that the choroid undergoes a rapid decrease in the more central area, which is possibly related to macular pathology, including macular hole, macular atrophy and choroidal neovascularization (CNV), which may occur in pathologic myopia. This speculation needs further longitudinal study to verify.

There have been many unresolved disputes regarding the relationship between age and choroidal thickness in children. The present study demonstrated that increasing age was associated with decreased choroidal thickness of all the nine sectors for the total population, which is consistent with previous studies in Asian children^21^,^22^,^23^,^29^,^30^. However, in white children, age has been found to be positively associated with subfoveal choroidal thickness^24^,^25^,^26^. In our relatively larger sample of children, when stratified by refractive errors, choroidal thickness was positively associated with age for emmetropes and hyperopes but was negatively associated with age for myopes. Thus, the discordance between Asian and white populations might be due to the different distribution of the refractive errors in the individuals recruited in the studies. This is because study samples from Asia usually have a higher proportion of individuals with myopia and therefore may have thinner choroids.

In the present study, we further found that a spherical equivalent of −2.00 D was a point below which the choroid no longer associates with increasing age. The two separate phenomena, the thickening of the choroid related to normal ocular growth and development and the thinning of the choroid related to myopia development and progression, have been implicated in the longitudinal study by Scott *et al*.^28^. The thickening of the choroid with age in childhood may reflect normal growth in the vascular and connective tissue structure of the choroid, which may function to slow eye growth during development either by regulating the scleral growth factors or by acting as a mechanical buffer to limit axial elongation^6^,^41^. The disappearance of the positive effect of age in myopes equal to or less than −2.00 D might indicate the disappearance of the protective effect of normal choroidal growth against rapid axial elongation, which is typically associated with myopia progression. Axial elongation may, in turn, influence the physical properties of the choroidal vessels^42^. These intricate interactions between choroidal growth and axial changes have some implications for the mechanisms underlying myopic eye growth and for the retinopathy that is often associated with high myopia.

Among hyperopes, girls had a significantly thicker choroid than boys, but this significance disappeared after adjusting for age, AL and spherical equivalent, which was consistent with most of the previous studies^19^,^21^,^24^,^30^. However, in adult studies, men have been found to have thicker choroid than women^14^,^16^,^17^. This discrepancy might be explained by the different onset time and duration of puberty in girls and boys. Boys usually have later onset but longer duration and larger growth amplitude of puberty^43^,^44^. Li *et al*. compared the choroidal thickness between boys and girls based on the stratification of puberty stages and found thicker choroidal thickness in girls with more advanced pubertal development. However, in that study, a smaller proportion of boys aged 11 to 12 years old had entered or completed puberty, which might have led to potential bias^19^. Future studies including older children and evaluating the relationship between gender and choroidal thickness stratified on pubertal development might be helpful for elucidating this issue.

A negative correlation between choroidal thickness and AL has been well established in previous studies in both children and adults^8^,^9^,^13^,^17^,^21^,^24^,^25^,^31^,^34^,^38^. Our results demonstrated that a 1 mm increase in AL was associated with a 16 μm decrease in central foveal choroidal thickness in the total population, and the associated decrease ranged from 9 μm to 24 μm in individuals with various refractive statuses. Choroidal thickness was also found to be strongly associated with spherical equivalent, with a decrease in choroidal thickness of 7.6 μm for each diopter of myopic shift, which was consistent with previous studies^23^,^25^,^31^.

IOP and BMI were found to be significantly positively related to choroidal thickness in the total population. There have been studies indicating changes of IOP as a potential mechanism in the thermoregulation of the retina by the choroid, although this is not universally accepted^1^. Future studies are needed to further investigate the mechanisms and implications underlying the association between IOP and choroidal thickness. The finding of BMI being related to choroidal thickness suggested that hormones might be important for choroidal thickness. However, the results showed associations between choroidal thickness and IOP as well as BMI under different refractive status that are hard to interpret. For example, IOP was significantly associated with choroidal thickness for hyperopes but not for myopes, while BMI was significantly related to choroidal thickness for myopes but not for hyperopes. Since IOP and BMI are potential influencing factors, the findings presented here might provide some clues for future studies.

There are several limitations in our study. First, as this is a cross-sectional study, the causation of the association we found, as well as the exact growth rates in different age intervals, cannot be determined. Future longitudinal investigations are necessary. Second, as we examined only Chinese children aged 6 to 19 years old, the choroidal thickness in children of other races and those younger than 6 years old was not determined. Studies by Scott *et al*. and Nagasawa *et al*. demonstrated a marked change of choroidal thickness between pre-school children and school children^21^,^24^. Future studies including a larger sample size of younger children are needed to determine the profile of their choroid growth. Last but not the least, the variation in demographic and biometric variables examined in our study only account for a relatively small percentage (27%) in the variance of choroidal thickness in this population and was even smaller in emmetropes (5.8%) and hyperopes (6.4%). Future studies examining a wider range of ocular and demographic parameters may help us better understand the factors influencing choroidal thickness in Chinese children.

In conclusion, we found a greater difference in the more central region between myopes and emmetropes and an evenly distributed difference across the macular region between hyperopes and emmetropes, except for the nasal sector. Choroidal thickness increased with age in emmetropia and mild myopia with spherical equivalent > −2.00 D, but not in children with spherical equivalent ≤ −2.00 D, suggesting that the protective effect of physiologic choroidal growth with age against rapid axial elongation disappeared while axial elongation becomes the dominant determinant of choroidal thickness among children with myopia worse than −2.00 D.

## Methods

### Setting and Participants

School children aged 6 to 19 years from a total of 12 primary and middle schools in Songjiang and Jiading District in Shanghai were enrolled with cluster sampling. All of the children who participated understood the study protocol, and their parents or legal guardians signed written informed consents. Children with ocular pathology, including amblyopia (best corrected visual acuity [BCVA] < 20/25) and strabismus, previous ocular surgery and poor quality SS-OCT scans were excluded. The tenets of the Declaration of Helsinki were followed, and the Institutional Review Board of Shanghai General Hospital, Shanghai Jiao Tong University approved the study.

The investigation was conducted within the schools from November 2015 to January 2016. The research team consisted of one ophthalmologist, five optometrists, two public health physicians and two nurses.

### Research Methods

The age and gender of all the participants were recorded according to state-issued identification cards. The participants’ height and weight were first measured at the research site. Then, all of the participants underwent comprehensive ophthalmic examinations, including visual acuity, AL, intraocular pressure (IOP), slit-lamp examination, cycloplegic refraction and SS-OCT imaging.

Visual acuity was tested at a 4-meter distance using a retro-illuminated Early Treatment of Diabetic Retinopathy Study (ETDRS) chart. AL was measured using IOL Master (version 5.02, Carl Zeiss Meditec, Oberkochen, Germany), and IOP was measured with a non-contact tonometer (model NT-4000, Nidek Inc., Fremont, CA, USA). Cycloplegia was achieved by administering one drop of topical 0.5% proparacaine (Alcaine, Alcon), followed by two drops of 1% cyclopentolate (Cyclogyl; Alcon, Fort Worth, TX, USA), 5 minutes apart in each eye. Pupil size and light reflex were examined 30 minutes after the last drop of cyclopentolate, and cycloplegia was deemed complete if the pupil dilated ≥ 6 mm and light reflex was absent. Refraction was performed using a desk-mounted auto-refractor (model KR-8900, Topcon, Tokyo, Japan). Children with presenting visual acuity lower than 20/25 were assessed for subjective refraction.

**Figure 4:**
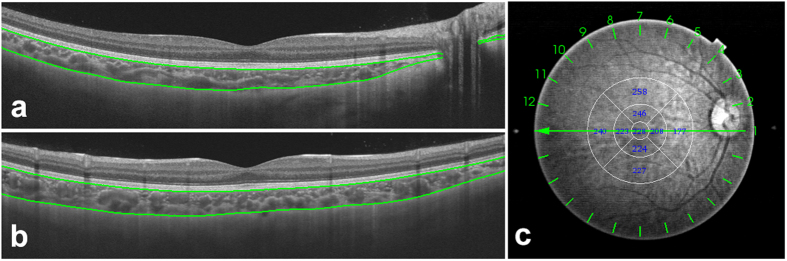
Choroidal thickness map of a healthy 9-year-old girl obtained by SS-OCT. (**a**) Choroidal thickness, measured as the distance between the Bruch membrane and the choroid-sclera interface, horizontally (**a**) and vertically (**b**). (**c**) The ETDRS grid was applied to the map, and the mean choroidal thickness was obtained for each sector.

SS-OCT (model DRI OCT-1 Atlantis; Topcon), with an axial resolution of 8 μm and a transverse resolution of 10 μm, was adopted to measure the choroidal thickness. This adopted OCT uses a 1050-nm-wavelength light source and has a scanning speed of 100,000 A-scans per second. The 12-line radial scan pattern with a resolution of 1024 × 12 was used. Each image was an average of 32 overlapped consecutive scans focused on the fovea, covering an area of 12 mm × 9 mm. The measurement was conducted from 10 AM to 3 PM to reduce the impact of diurnal variation^45^. Built-in software was used to segment layers and construct topographic maps. All acquired images were inspected, and if automatic segmentation errors occurred or resulted in measurement artifacts, manual segmentation was performed. Choroidal thickness was defined as the distance between the Bruch membrane and the choroid-sclera interface (Fig. 4a and b). The ETDRS grid was used for the choroidal thickness map (Fig. 4c), and the mean regional thicknesses were calculated for the nine sectors of the grid^13^,^21^,^31^. The diameters for the central foveal circle, parafoveal circle and perifoveal circle were 1 mm, 3 mm and 6 mm, respectively, and they were further divided into superior, inferior, temporal, and nasal quadrants.

### Statistical Analyses

A database was created using Epidata 3.1. All data was doubly-entered independently by two trained staff members and all discrepancies were adjudicated. The statistical analyses were conducted using IBM SPSS statistics version 20.0 (IBM Co., Armonk, NY, USA). Only the data for the participants’ right eyes was included for statistical analysis.

Body mass index (BMI) was calculated using the formula: weight (kg)/[height (m)]^2^. Spherical equivalent was used to classify refractive status and was obtained as follows: spherical equivalent = S + C/2. Myopia, emmetropia and hyperopia were defined as spherical equivalent ≤ −0.5 diopters (D), −0.5 D < spherical equivalent < +0.5 D, spherical equivalent ≥ +0.5 D, respectively^31^,^46^. Myopia was further categorized into high myopia, moderate myopia and mild myopia, which was defined as spherical equivalent ≤ −5.0 D, −5.0 D < spherical equivalent ≤ −3.0 D, −3.0 D < spherical equivalent ≤ −0.5 D, respectively.

The parameters were presented as the mean ± standard deviation (SD) for continuous variables and as rates (proportions) for categorical data. Intergroup differences were tested by t test (between genders) or variance analysis (among refractive groups). The categorical variables were compared with chi-square tests. The choroidal thickness at different regions were compared using repeated-measures analysis of variance (RM-ANOVA) by testing for sphericity. When the sphericity assumption was violated, the Greenhouse-Geis test was used. The Bonferroni method was used to adjust for comparisons across these post hoc tests. Simple linear regression was used to display the association of choroidal thickness in different regions with age for myopes, emmetropes and hyperopes. Multiple regression analysis was performed to explore independent associated factors for choroidal thickness under different refractive status. Statistical significance was set at p < 0.05 (two tailed). The sample size of each refractive group was calculated as presented in Supplementary Material S1.

## Additional Information

**How to cite this article**: Xiong, S. *et al*. Choroidal Thickness in 3001 Chinese Children Aged 6 to 19 Years Using Swept-Source OCT. *Sci. Rep.* **7**, 45059; doi: 10.1038/srep45059 (2017).

**Publisher's note:** Springer Nature remains neutral with regard to jurisdictional claims in published maps and institutional affiliations.

## Supplementary Material

Supplementary Material S1

## Figures and Tables

**Table 1:** General Characteristics of the 3001 Participants and Comparison Between Different Refractive Groups.

Parameters	Total N = 3001	Range	Myopes N = 1548	Range	Emmetropes N = 441	Range	Hyperopes N = 1012	Range	*p* value
	Mean ± SD^a^		Mean ± SD^a^		Mean ± SD^a^		Mean ± SD^a^		
Boys, No. (%)	1555 (51.8)	—	765 (49.4)	—	226 (51.2)	—	564 (55.7)	—	0.007^b^
Age, years	11.7 ± 3.4	6~19	13.5 ± 3.02	6.3~19.4	11.0 ± 2.9	6.3~18.4	9.2 ± 2.4	6.3~18.0	<0.001^c^
Height, cm	148.7 ± 17.2	109.0~193.0	157.6 ± 14.2	117.0~193.0	146.0 ± 15.2	112.0~186.0	136.3 ± 13.8	109.0~184.5	<0.001^c^
Weight, kg	43.4 ± 16.5	16.5~130.0	50.7 ± 15.9	20.0~130.0	41.1 ± 14.36	16.5~105.0	33.2 ± 12.0	17.0~87.0	<0.001^c^
BMI, kg/m^2^	18.9 ± 3.8	10.8~40.6	20.0 ± 3.9	12.4~40.6	18.7 ± 3.6	11.3~31.4	17.33 ± 3.19	10.84~30.48	<0.001^c^
IOP, mmHg	16.5 ± 2.6	9~25	16.7 ± 2.5	9~25	16.7 ± 2.5	10~24	16.3 ± 2.6	9~24	0.001^c^
AL, mm	24.04 ± 1.31	20~29	24.90 ± 1.10	22~29	23.48 ± 0.75	21~26	22.97 ± 0.76	20~27	<0.001^c^
Spherical equivalent, D	−1.20 ± 2.48	−11.38~8.38	−3.08 ± 2.00	−11.38~−0.5	0.03 ± 0.25	−0.38~0.38	1.14 ± 0.70	0.50~8.38	<0.001^c^

BMI: body mass index; AL: axial length; IOP: intraocular pressure; SD: standard deviation; D: diopter.

^a^Except where noted otherwise.

^b^Statistical significance was tested using Chi-Square tests.

^c^Statistical significance was tested using variance analysis.

**Table 2:** Central Foveal Choroidal Thickness of Each Age Interval.

Age, yrs	N	Mean ± SD, μm	Range, μm	Percentiles (25, 50, 75), μm
6	51	272 ± 61	156~445	241, 267, 308
7	276	283 ± 63	145~538	238, 276, 325
8	309	269 ± 61	115~457	226, 266, 306
9	403	257 ± 58	113~451	216, 250, 293
10	326	244 ± 59	116~426	200, 241, 283
11	260	239 ± 58	89~441	201, 232, 275
12	160	246 ± 59	95~404	202, 242, 283
13	249	229 ± 59	107~455	189, 225, 268
14	222	229 ± 63	92~418	179, 229, 267
15	134	218 ± 69	100~430	171, 207, 263
16	260	220 ± 71	77~452	167, 208, 266
17	206	234 ± 77	68~481	176, 228, 279
18–19	145	218 ± 69	69~424	165, 215, 266
Total	3001	245 ± 66	68~538	198, 242, 285

**Table 3:** Multiple Regression Analysis of Associations With Central Foveal Choroidal Thickness for Myopes, Emmetropes and Hyperopes.

Variables	Myopes		Emmetropes		Hyperopes	
	Estimate (95%CI)	*p*	Estimate (95%CI)	*p*	Estimate (95%CI)	*p*
Age	1.091 (−0.004, 2.187)	0.051	3.859 (1.815 to 5.902)	<0.001	1.569 (−0.131 to 3.269)	0.070
Gender	−3.322 (−9.497 to 2.852)	0.291	0.959 (−10.626 to 12.544)	0.871	5.880 (−2.084 to 13.844)	0.148
BMI	1.061 (0.253 to 1.870)	0.010	−1.183 (−2.851 to 0.485)	0.164	0.896 (−0.400 to 2.192)	0.175
AL	−15.861 (−19.949 to −11.772)	<0.001	−8.955 (−16.907 to −1.004)	0.027	−15.314 (−21.052 to −9.577)	<0.001
Spherical equivalent	3.849 (1.693 to 6.005)	<0.001	34.234 (12.525 to 55.943)	0.002	6.324 (0.666 to 11.983)	0.029
IOP	0.614 (−0.488 to 1.715)	0.275	0.850 (−1.218 to 2.918)	0.420	1.616 (0.231 to 3.001)	0.022

Adjusted for all variables listed. R^2^ for myopes: 0.135; R^2^ for emmetropes: 0.064; R^2^ for hyperopes: 0.062.

BMI: body mass index; AL: axial length; IOP: intraocular pressure; CI: confidence interval; D: diopter.

**Table 4:** Multiple Regression Analysis of Associations With Central Foveal Choroidal Thickness for high, moderate and mild myopes.

Variables	High Myopes (n = 269)		Moderate Myopes (n = 431)		−2.00 D~−3.00 D (n = 313)		−2.00 D~−1.25 D (n = 248)		−1.25 D~−0.50 D (n = 287)	
	Estimate (95%CI)	*p*	Estimate (95%CI)	*p*	Estimate (95%CI)	*p*	Estimate (95%CI)	*p*	Estimate (95%CI)	*p*
Age	−1.656 (−4.445 to 1.132)	0.243	−0.265 (−2.359 to 1.829)	0.804	0.799 (−1.902 to 3.499)	0.561	3.232 (0.516 to 5.949)	0.020	3.476 (1.001 to 5.951)	0.006
Gender	−5.849 (−19.236 to 7.537)	0.390	1.132 (−10.505 to 12.769)	0.849	−5.466 (−20.269 to 9.338)	0.468	−0.086 (−26.006 to 6.267)	0.229	−2.071 (−16.744 to 12.602)	0.781
BMI	1.568 (0.016 to 3.119)	0.048	1.927 (0.346 to 3.508)	0.017	1.472 (−0.540 to 3.483)	0.151	0.818 (−1.248 to 2.885)	0.436	−1.807 (−3.874 to 0.259)	0.086
AL	−13.604 (−22.029 to −5.180)	0.002	−10.408 (−18.149 to −2.667)	0.009	−24.236 (−34.070 to −14.403)	<0.001	−17.043 (−28.365 to −5.722)	0.003	−17.754 (−27.234 to −8.274)	<0.001
Spherical equivalent	5.525 (−0.069 to 11.118)	0.053	1.512 (−8.680 to 11.705)	0.771	−4.072 (−26.606 to 18.463)	0.722	2.250 (−33.224 to 37.723)	0.901	3.550 (−26.396 to 33.497)	0.816
IOP	0.805 (−1.602 to 3.213)	0.511	0.224 (−1.868 to 2.315)	0.834	0.976 (−1.740 to 3.693)	0.480	0.788 (−2.145 to 3.722)	0.529	0.526 (−2.042 to 3.094)	0.687

Adjusted for all variables listed. R^2^ for high myopes: 0.129; R^2^ for moderate myopes: 0.033; R^2^ for −2.00 D~−3.00 D: 0.083; R^2^ for −2.00 D~−1.25 D: 0.062; R^2^ for −1.25 D~−0.50 D: 0.075.

BMI: body mass index; AL: axial length; IOP: intraocular pressure; CI: confidence interval; D: diopter.

High myopia and moderate myopia was defined as spherical equivalent ≤ −5.0 D, −5.0 D < spherical equivalent ≤ −3.0 D, respectively.
