# Supplementary material for: Choroidal Thickness in 3001 Chinese Children Aged 6 to 19 Years Using Swept-Source OCT
Source: Sci Rep. 2017 Mar 22;7:45059. doi: 10.1038/srep45059 (PMC5361145; doi:10.1038/srep45059)
Supplement: Supplementary Material S1 [file srep45059-s1.pdf]

Supplementary Material S1:

Choroidal Thickness in 3001 Chinese Children Aged 6 to 19 Years Using Swept-Source OCT

Shuyu Xiong[1,2],Xiangui He[1,3 *], Junjie Deng[1,2],Minzhi Lv[1],Jiali Jin[1],Sifei Sun[4],Yaochun Xia[5],Jianfeng Zhu[1], Haidong Zou[1,2],Xun Xu[1,2 *]

1. Department of Preventative Ophthalmology, Shanghai Eye Disease Prevention and Treatment Center, Shanghai Eye Hospital, Shanghai 200040, China
2. Department of Ophthalmology, Shanghai General Hospital, Shanghai Jiao Tong University, Shanghai 200080, China
3. School of Public Health, Fudan University, Shanghai 200032, China
4. Jiading Center for Disease Prevention and Control, Shanghai 201901, China
5. Songjiang Center for Disease Prevention and Control, Shanghai 201620, China

Supplementary Material S1:

We calculated the sample size using the following formula:

$$\mathbf{n = \Psi^2 \left(\sum (S_i^2) / K \right) / \left[\sum (X_{imean} - X_{mean})^2 / (K-1) \right]}$$

1) α= 0.05, β= 0.10;

2) K: number of groups; in our study, three groups (myopes, emmetropes and hyperopes) were included; K = 3;

3) Ψ: $\Psi_{\alpha, \beta, K-1, \infty}$=2.52;

4) X_{imean}、S_i: represent the mean value and standard deviation of the group i (i =1, 2, 3…). In the present study, the mean value and standard deviation was estimated on the basis of previous study by Jin et al: Jin, P. et al. Choroidal and Retinal Thickness in Children With Different Refractive Status Measured by Swept-Source Optical Coherence Tomography. American journal of ophthalmology, doi:10.1016/j.ajo.2016.07.003 (2016).

The above calculation comes up with a number of 44 participants in each group. When we take the lost of follow-up rate into consideration (set as 20%), each group needs a minimum number of 53 participants. In our study, we enrolled 1548 myopes, 441 emmetropes and 1012 hyperopes, which are larger than the estimated sample size.
